# Mechanobiology of 3D cell confinement and extracellular crowding

**DOI:** 10.1007/s12551-024-01244-z

**Published:** 2024-10-23

**Authors:** Gabriela Da Silva André, Céline Labouesse

**Affiliations:** Macromolecular Engineering Laboratory, Department of Mechanical and Process Engineering, ETH Zurich, 8092 Zurich, Switzerland

**Keywords:** Confinement, Mechanotransduction, Compressive stresses, Macromolecular crowding, Organoid, Tumor spheroid

## Abstract

Cells and tissues are often under some level of confinement, imposed by the microenvironment and neighboring cells, meaning that there are limitations to cell size, volume changes, and fluid exchanges. 3D cell culture, increasingly used for both single cells and organoids, inherently impose levels of confinement absent in 2D systems. It is thus key to understand how different levels of confinement influences cell survival, cell function, and cell fate. It is well known that the mechanical properties of the microenvironment, such as stiffness and stress relaxation, are important in activating mechanosensitive pathways, and these are responsive to confinement conditions. In this review, we look at how low, intermediate, and high levels of confinement modulate the activation of known mechanobiology pathways, in single cells, organoids, and tumor spheroids, with a specific focus on 3D confinement in microwells, elastic, or viscoelastic scaffolds. In addition, a confining microenvironment can drastically limit cellular communication in both healthy and diseased tissues, due to extracellular crowding. We discuss potential implications of extracellular crowding on molecular transport, extracellular matrix deposition, and fluid transport. Understanding how cells sense and respond to various levels of confinement should inform the design of 3D engineered matrices that recapitulate the physical properties of tissues.

## Introduction

In most tissues, the microenvironment exerts substantial constraints on cell shape and cell volume, confining cells to a certain degree and forcing migrating cells to squeeze through constricted spaces (Lämmermann and Germain 2014; McGregor et al. 2016). Confinement is imposed by high extracellular matrix (ECM) density and/or high cell density, in particular in 3D tissue contexts or in 3D in vitro culture. While the importance of the ECM and of cell–cell communication is well described, their function is rarely looked at through the lens of the provided confinement. 3D confinement is important for several mechanosensitive pathways that are intrinsically sensitive to cell shape, cell volume, and compressive stresses (Saraswathibhatla et al. 2023). Furthermore, the extracellular crowding associated with confined spaces restricts molecular transport in the pericellular space, impacting cellular communication (Li and Kumacheva 2018; Raghunath and Zeugolis 2021; van Niel et al. 2022). In this review, we synthesize the current understanding of mechanobiology of cell confinement and extracellular crowding, in the context of single cells, organoids, and tumor spheroids. Our aim is to provide new insights on confinement, which will inform the design of engineered biomaterials for models of healthy and diseased tissues.

Many mechanosensitive pathways were first mechanistically described using two-dimensional (2D) cell culture assays. In standard 2D cell culture, there is minimal confinement imposed on cells. Mesenchymal cells, e.g., fibroblasts, are usually cultured as single cells on an adherent surface with no limitation on the cell volume or on the exchange between the cell and the surrounding milieu. In monolayer cultures, e.g., of epithelial cells, the cell density can provide some constraints to cell volume that are akin to contact-inhibition in tissues (Gudipaty et al. 2017; Miroshnikova et al. 2018), but the apical surfaces remain free. Cell or colony area can be further constrained by using micropatterned substrates, resulting in specific patterns of traction forces and of signaling, which in turn create patterns of proliferation or differentiation (Nelson et al. 2005; Xue et al. 2018). However, in all these examples, the apical surfaces of cells remained unconfined, and there is no limitation to molecular exchange with the surrounding milieu.

2D culture systems cannot accurately reproduce in vivo levels of confinement, instead three-dimensional (3D) systems could be used to create more realistic tissue models. The last decade has seen a rapid growth in the use of such 3D systems in which cells or cell aggregates are encapsulated into ECM-mimicking engineered matrices (Caliari and Burdick 2016). Such systems have enabled the exploration of 3D mechanosensitive pathways involved in morphogenesis, organoid development, and tumor progression (Baker and Chen 2012; Li and Kumacheva 2018; Kratochvil et al. 2019; Saraswathibhatla et al. 2023). In these 3D systems, the surrounding microenvironment presents defined constraints on cell shape and volume and on molecular exchange within the tissue constructs. While many of the discovered mechanosensitive pathways are conserved in 3D, their sensitivity to specific mechanical and physical stimuli can diverge from what has been observed in 2D culture (Saraswathibhatla et al. 2023). The dimensionality of the cell and tissue models thus matters for activation of mechanosensitive pathways. This sensitivity in 3D could potentially be attributed to the confinement felt in 3D microenvironments and would be expected to depend on scaffold properties (stiffness, viscoelasticity, porosity, and degradability), although the specific dependencies are not straightforward to infer.

**Fig. 1:**
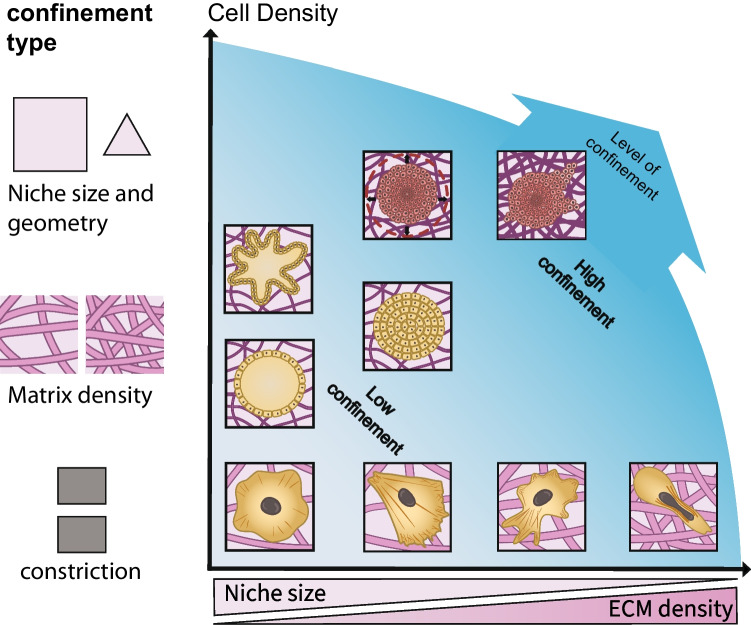
Matrix density and cell density both participate to impose confinement on cells. (Left) We discuss different types of confinement, imposed either by microwells or microniches of defined size and geometry, by hydrogels/engineered matrices at different polymer densities (i.e., different stiffness), or constrictions through which cells can squeeze through, typically made with PDMS pillars. (Right) We distinguish different levels of confinement (color gradient), based on cell and matrix density and on the deformation imposed on cells. The *x*-axis represents the confinement imposed by matrix density or equivalently by manufacturing cell niches of increasingly smaller sizes, which both limit cell volume or even deform the cell and nucleus (highest level of confinement). The *y*-axis represents the confinement imposed by increasing cell density at a constant volume, as seen for cell aggregates (organoids, embryoid bodies, and tumor spheroids). Cancer cells are shown in red

In this review, we define different levels of confinement (Fig. 1) and provide an overview of their known effects on cell viability, cell mechanics, and cell function. We first look at low levels of confinement (i.e., limitation to volume and volume growth). We discuss how seeding cells or embryoid bodies in microwells impact viability, function, and activation of mechanosensitive pathways. We also consider 3D encapsulation of cells and organoids in hydrogel-based elastic scaffolds, where scaffold stiffness must be optimized for survival and growth. We cover how scaffold viscoelasticity can modulate the level of confinement and mechanosignaling and ultimately facilitate organoid differentiation. We then describe intermediate levels of confinement, imposed by high matrix density combined with abnormally high cell density in tumors and tumor spheroids. The intermediate confinement results in high gradients of compressive stresses and of cell volume. We move on to discuss how high levels of confinement that significantly deform not only the cells but also their nucleus, trigger specific adhesion-independent migration modes. In the case of tumors, high levels of confinement can trigger cell softening and an unjamming phenomenon that drives tumor invasion. Finally, we discuss the impact of extracellular crowding on macromolecular transport in the pericellular space, regulating ECM deposition, vesicular transport, and fluid transport in tumors. We close by suggesting some open questions for the field.

## Low levels of confinement activate multiple mechanosensitive pathways

### Microwell confinement modulates mechanosensitive signaling in cells and embryoid bodies

Seminal work on cell size and attachment in 2D has shown that adherent cells do not survive if confined to a too small area (Chen et al. 1997). Cell spreading and integrin engagement at cell–matrix adhesions are required for proliferation, survival, and stem cell differentiation (Humphrey et al. 2014). Since cell–matrix adhesion complexes are inherently different in 2D and 3D, the role of focal adhesions in sensing cellular confinement in 3D is of central interest (Cukierman et al. 2001). As the mechanobiology field has moved increasingly to 3D microenvironments, recent studies have addressed whether low levels of confinement that restrict cell volume, volume growth, and shape changes would impact downstream cell function, in particular survival and proliferation in 3D (Baker and Chen 2012).

To determine how confinement could impact cell viability and cell mechanotransduction, cells were cultured in single cell microniches of defined size, shape, and aspect ratio. These microniches have been microfabricated or molded in hydrogels (Bao et al. 2017; Dudaryeva et al. 2021) or in elastomers (Jain and Vogel 2018) and functionalized with adhesion motifs. The microniches were specifically designed to limit cell volume without deforming the nucleus (low levels of confinement) and were stiff (*E* ≥ 30 kPa). In these low confinement conditions, the viability of human mesenchymal stem cells (hMSCs) remained high (> 80% after 3 days) even in the smaller niches (Dudaryeva et al. 2021). Only in softer (*E* ~ 6 kPa) and small niches was viability lower (67%). Lower viability correlated with lower Yes-associated protein 1 (YAP) activation (Dudaryeva et al. 2021). In stiff microniches that were closed with hydrogel lids, hMSCs filled the whole volume of the niche (Bao et al. 2017). In these cases, the number of stress fibers increased with decreasing niche size up to a point (roughly 1.7 times the volume of cells in suspension). Concurrently, focal adhesion maturation, myosin activity, and YAP nuclear activity increased in line with the increase in stress fibers (Bao et al. 2017). Taken together, these results indicate that at low levels of confinement the confinement volume itself is not a limiting factor for cell viability. Rather, the level of mechanoactivation, determined in part by niche stiffness, is critical for cell survival.

Activation of mechanosensitive pathways is essential not only for survival, but also for many specific cell functions. We here consider the case of macrophages’ inflammatory response in 3D confinement. Macrophages have been cultured under low confinement, on small microwells (20–30 µm diameter), small micropatterned islands (Jain and Vogel 2018), or in the void space of a microporous annealed scaffold (Liu et al. 2023b). Contrary to what was seen in hMSCs in microniches, 2D confinement of macrophages led to lower overall F-actin levels, lower myocardin-related transcription factor A and serum response factor (MRTF-A/SRF) pathway activation, and lower expression of histone deacetylase 3 (HDAC3) (Jain and Vogel 2018). These transcription factors and chromatin modifiers are involved in driving the late inflammatory response upon lipopolysaccharide (LPS) stimulation. LPS stimulation of 2D confined macrophages indeed resulted in a lower activation of late pro-inflammatory factors (Jain and Vogel 2018). 3D confinement of macrophages similarly resulted in significantly lower levels of pro-inflammatory markers, such as IL6 and CD86 and genes *IL6*, *iNOS*, and *CXCL9* (Jain and Vogel 2018; Liu et al. 2023b). Therefore, spatial confinement appears to be an important factor impacting macrophage inflammatory response (Jain and Vogel 2018; Liu et al. 2023b, a), although whether F-actin, MRTF-A, or HDAC3 are involved in 3D confinement still needs to be determined.

Embryoid bodies have also been cultured in microwells of defined size and shape (Hwang et al. 2009; Mohr et al. 2010; Sen et al. 2021). Limiting total volume did not adversely affect formation or viability of the embryoid bodies, but did impact subsequent cardiac differentiation (Hwang et al. 2009; Mohr et al. 2010). Smaller volumes (100–150 µm) promoted endothelial cell differentiation, while larger volumes (~ 300 µm) promoted cardiomyocyte differentiation and formation of beating colonies (Hwang et al. 2009; Mohr et al. 2010). Here, Wnt signaling was implicated in the size-dependent fate patterning of the embryoid bodies, specifically Wnt5a and Wnt11 (Hwang et al. 2009). Therefore, 3D geometrical confinement can influence signaling patterns in embryoid bodies and impact differentiation programs.

### Encapsulation in elastic hydrogels limits volume dynamics, organoid viability, and maturation

In the examples above, the cell or embryoid body confinement volume was defined by the design parameters of the engineered matrices, making it easier to investigate the role of stiffness and volume independently. Low levels of confinement also occur when encapsulating cells in hydrogels, in particular in nanoporous hydrogels. The confinement volume here is not fixed, but depends on the stiffness, polymer density, and deformability of the hydrogel material (Caliari and Burdick 2016). In bulk elastic hydrogels, e.g., with a poly(ethylene glycol) (PEG) or gelatin methacrylate (GelMA) backbone, the pore size directly correlates with the density of cross-linking points and therefore with stiffness (Nichol et al. 2010; Mabry et al. 2016). In these cases, stiffer and denser matrices generally decreased cell viability (Nichol et al. 2010).

**Fig. 2:**
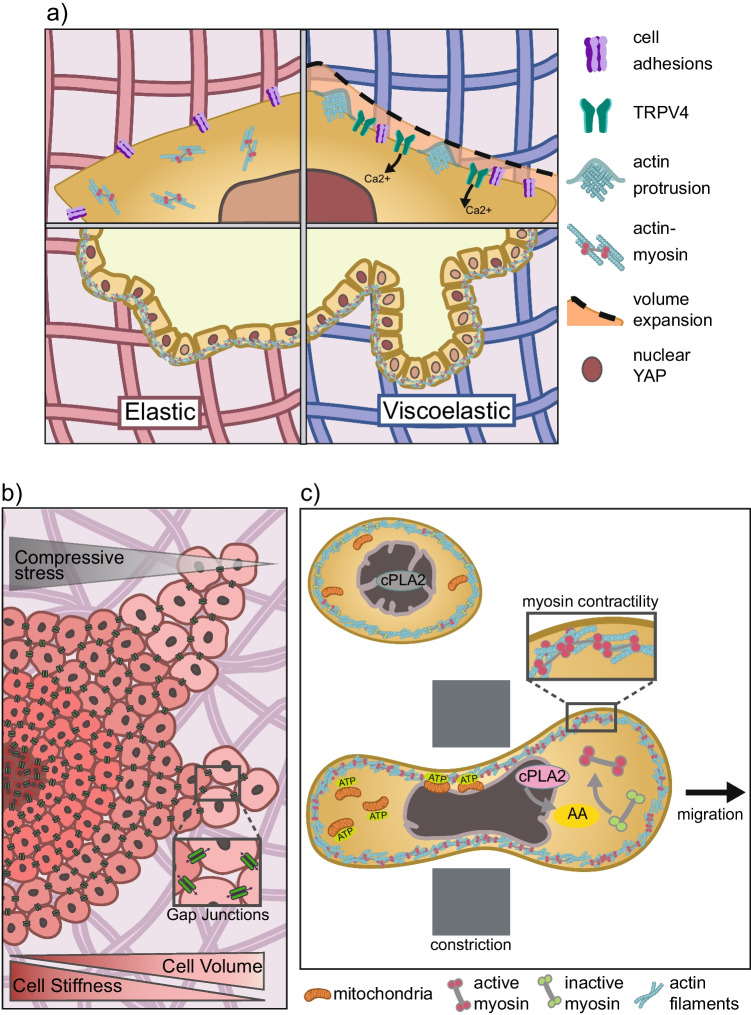
**a** Pathways involved in sensing confinement in single cells (top half) or in organoids (bottom half). Elastic matrices or slow-relaxing viscoelastic matrices (red) and fast-relaxing viscoelastic matrices (purple) both mediate confinement. In elastic matrices, adhesions and contractility are required for cell survival under confinement. In viscoelastic matrices, stretch-activated channels are additionally involved in mediating volume expansion. Actin protrusions also mediate cell shape dynamics in deformable scaffolds. In intestinal organoids, additionally, the importance of the YAP pathway has been observed. YAP is not highly activated enough in elastic matrices, but in slow-relaxing viscoelastic matrices, YAP is activated in a heterogeneous manner (cell size dependent), enabling fate patterning and budding. **b** Tumor spheroid confined in a dense ECM corresponding to intermediate levels of confinement. The confinement leads to a gradient of compressive stresses that increases towards the center. This is accompanied by a corresponding gradient in cell stiffness and an inverse gradient in cell volumes, with larger cell volumes found at the spheroid periphery. Volume changes under compressive stresses are mediated by fluid exchange through gap junctions. Cell softening at the periphery tends to drive protrusions and invasion into the stroma. **c** High levels of confinement deform the cell and nucleus. Nuclear indentation by constrictions leads to unfolding of the nuclear envelope, release of calcium ions (not shown), activation of the phospholipase cPLA2 at the nuclear envelope, and release of arachidonic acid (AA) into the cytoplasm. The combined effect of Ca^2+^ and AA increases cell contractility that powers amoeboid migration. In migrating cells under confinement, a relocalization of mitochondria is observed around the nucleus and in the posterior part of the cell, leading to localized ATP production. Figure adapted from (Venturini et al. 2020)

A similar decrease in cell viability has been found in some cases when cell aggregates or organoids were encapsulated in hydrogels (Cruz-Acuña et al. 2017), although with a dependence on cell type or origin. Organoids are model organs that recapitulate stages of development and are typically cultured in suspension, or in Matrigel. Matrigel provides a confining biophysical environment as well as biochemical factors, but has a variable composition and concentration of growth factors (Aisenbrey and Murphy 2020). To replace Matrigel with a more controllable system, researchers have embedded organoids in defined hydrogels that provide adhesion cues, polarity cues, and a low level of confinement (Gjorevski et al. 2016, 2022; Cruz-Acuña et al. 2017; Indana et al. 2021). For many organoids, the beneficial effect of encapsulating them in a hydrogel critically depends on defining the optimal hydrogel stiffness, close to the stiffness of the in vivo tissue context. Kidney organoids encapsulated in soft gels (G´ ≤ 0.7 kPa) differentiated better and had fewer aberrant phenotypes (Garreta et al. 2019; Geuens et al. 2021; Ruiter et al. 2022). On the other hand, liver organoids formed best in stiffer gels of G´ ~ 1.3 kPa rather than in softer gels (Sorrentino et al. 2020). Pancreatic ductal carcinoma organoids formed best at E between 3 and 20 kPa, with the stiffest hydrogels eliciting the highest YAP/TAZ mechanosignaling (Below et al. 2022). The optimal stiffness appears to be highly dependent on the origin of the cells and on the stage of organoid formation (Gjorevski et al. 2016, 2022; Cruz-Acuña et al. 2017). For example, mouse intestinal stem cell colony formation formed readily and remained viable in matrices up to G´ ~ 1.3 kPa, and matrix degradability was detrimental to stem cell self-renewal and colony polarity (Gjorevski et al. 2016). However, prolonged compression due to a stiff confining matrix decreased YAP activation over time and delayed intestinal organoid morphogenesis (Gjorevski et al. 2016). In contrast, for human intestinal organoid studies, stiff elastic matrices (G´ ≥ 300 Pa compared with G´ < 100 Pa for Matrigel) decreased organoid survival while matrix degradability improved survival (Cruz-Acuña et al. 2017). A common finding is that cell contractility was required for intestinal organoid survival and expansion in 3D elastic hydrogels, as was YAP/TAZ transcriptional activity (Gjorevski et al. 2016; Cruz-Acuña et al. 2017). Overall, these results suggest that confinement of organoids in stiff elastic matrices has both positive and negative effects. Confinement can both promote YAP/TAZ signaling and cell contractility, but also induces adverse compression forces which block further development (Fig. 2a). The balance between these effects depends on each type of organoid and on the hydrogel used.

### Confinement in viscoelastic matrices: reciprocal feedback between cell volume expansion and stretch-activated ion channels

To partially release the confinement imposed by a stiff, elastic matrix, one option is to promote either hydrogel softening (via degradation) or stress relaxation (viscoelastic behavior, Box 1). Dynamic bonds (e.g., non-covalent cross-links) are often used to create viscoelastic hydrogels, for example ionic cross-links in alginate. These hydrogels have the additional advantage of decoupling stiffness and pore-size (Chaudhuri et al. 2016; Vining and Mooney 2017). Recent studies showed that the level of stress relaxation is an important factor in how cells respond to being confined in a 3D microenvironment (Chaudhuri et al. 2016, 2020; Darnell et al. 2018). Confinement sensing in these viscoelastic hydrogels depended not only on cell–matrix adhesions, but also on stretch-activated ion channels and actin protrusions. We briefly mention these below, but for a more detailed discussion on the role of stress relaxation in tissues, we refer the reader to other in-depth reviews (Chaudhuri et al. 2020; Saraswathibhatla et al. 2023).

**Figure:**
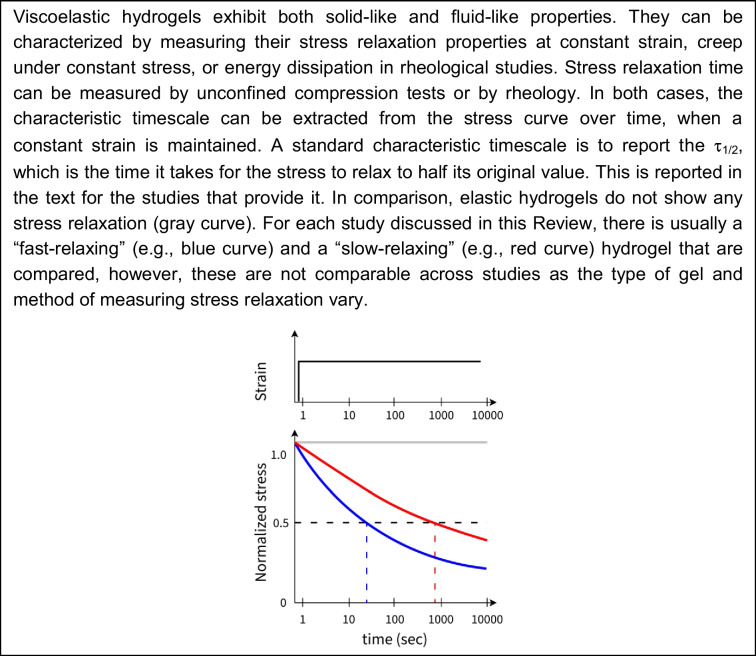
**Box 1**. Stress relaxation of viscoelastic matrices

A first observation is that cell viability increases in fast-relaxing hydrogels (i.e., more viscoelastic) compared with slow-relaxing hydrogels (i.e., more elastic, see Box 1) (Huang et al. 2023). hMSCs in slow-relaxing collagen hydrogels had higher rates of apoptosis triggered by hyperactivation of myosin contractility. These observations contrast with the requirement for myosin contractility for intestinal organoid survival in elastic matrices (Gjorevski et al. 2016; Cruz-Acuña et al. 2017), suggesting that there could be cell type or organoid-specific responses to confinement. In fast-relaxing hydrogels hMSCs also had higher nuclear YAP localization (Chaudhuri et al. 2016; Lee et al. 2019), more cell–matrix adhesions and long-term MSC chondrogenesis (Huang et al. 2023). A similar relation between stress relaxation rate and viability was found in human induced pluripotent stem cells (hiPSC) clusters: higher levels of apoptosis in slow-relaxing alginate matrices (τ_1/2_ ~ 1000 s), compared with fast-relaxing matrices (τ_1/2_ ~ 180 s) at equivalent stiffness (Indana et al. 2021). Only high concentration of adhesive binding sites (RGD peptides, ≥ 1.0 mM) partially rescued viability to > 70% and colony formation, similar to what has been observed in elastic matrices (Gjorevski et al. 2016; Cruz-Acuña et al. 2017; Indana et al. 2021). Interestingly, in the more viscoelastic matrices, low adhesion density but not hydrogel stiffness negatively impacted cell viability and stem cell pluripotency (Indana et al. 2021). Therefore, the stress relaxation properties, more so than the elastic modulus of the matrix, are critical for mechanosensing and downstream for cell viability, with fast-relaxing hydrogels promoting higher cell viability (Fig. 2a).

Cell sensing of 3D confinement is not only mediated by cell–matrix adhesion sites but also depends on mechanosensitive ion channels and on cortical actin dynamics, which control shape and volume changes. The viscoelasticity and deformability of the matrix on short timescales appeared to be important factors modulating the ability of cells to regulate cell volume (Lee et al. 2019; Nam et al. 2019; Chang et al. 2024). The activation of stretch-activated ion channels TRPV4, allowing an influx of Ca^2+^, facilitated cell volume expansion, which in turn led to higher TRPV4 expression, localization, and activation (Lee et al. 2019). This crosstalk of TRPV4 activation and cell volume expansion was found to be greater in fast-relaxing hydrogels and led downstream to increased osteogenesis of hMSCs (Fig. 2a). Other stretch-activated channels besides TRPV4 may also be involved (Nam et al. 2019; Chang et al. 2024). Overall, rapid changes in cell volume and/or cell shape are important mediators of mechanosensitive pathways regulating differentiation. Whether matrix properties impair or allow such morphological changes could be the critical point in determining if confinement has a positive or detrimental effect on stem cell survival, proliferation and differentiation.

### Partial release of confinement by ECM stress relaxation facilitates organoid differentiation

In organoids, cell volume and shape changes are constrained not only by matrix density but also by cell density, and both have a significant impact on differentiation (Fig. 1). Differentiating organoids appear to be more sensitive to confinement than the iPSC colonies they originate from, as only one of the tested matrix stiffness (G´ ~ 190 Pa) allowed for the formation of crypt-like buds in elastic matrices (Gjorevski et al. 2016, 2022). In addition, the cell crowding in high curvature regions (imposed by predefined niche geometry) led to the emergence of a localized YAP activation with higher nuclear YAP in the flat sides where cells were more spread, and cytoplasmic YAP in the small cells packed at the tips of the colony. The heterogeneous YAP activation was required for downstream patterning of cell fate (Gjorevski et al. 2022). YAP nuclear localization and fate patterning of Paneth cells could also be promoted by using stress-relaxing matrices. Tunable stress relaxation can be achieved by introducing a fraction of dynamic bonds in hydrogel cross-links (Ruiter et al. 2022; Chrisnandy et al. 2022). Stress relaxation (τ_1/2_ ~ 24 s) facilitated the budding of crypt compartments in intestinal organoids. Similarly, kidney organoids grown in a stress-relaxing hydrogel (τ_1/2_ ~ 3000 s) showed greater differentiation, polarization, and lumenization that their counterpart in slow-relaxing or elastic hydrogels (Geuens et al. 2021; Ruiter et al. 2022). Therefore, a partial release of confinement appears to be important for complete organoid differentiation (Fig. 2a).

## Intermediate levels of confinement lead to high compressive stresses in tumor spheroids

In the intestinal organoid examples described above, cell density is controlled by the balance of proliferation, differentiation, and apoptosis (Simons and Clevers 2011). In contrast to organoids, uncontrolled proliferation in tumor contexts leads to aberrantly high cell density which, together with a dense ECM, contributes to increasing confinement (Fig. 1). Confinement is known to play a crucial role in tumor development and progression (Le Maout et al. 2020; Almagro et al. 2022). The combination of constraints on tissue volume and of continued cell proliferation lead to an increased pressure in the cellular microenvironment and the emergence of high compressive solid stresses that can contribute to the malignancy and invasiveness of tumors (Nia et al. 2020).

The influence of solid mechanical stresses exerted by the environment on cancer cells is well described (Helmlinger et al. 1997; Montel et al. 2012; Alessandri et al. 2013). Solid stresses have been studied in vitro using hydrogels to encapsulate tumor-like cell aggregates called spheroids, thus creating a confining environment. The compressive stresses were measured by co-embedding fluorescent deformable beads (Dolega et al. 2017; Girardo et al. 2018; Taubenberger et al. 2019; Zhang et al. 2023). Solid stress build-up resulted in spheroid growth inhibition and the formation of a necrotic spheroid core (Helmlinger et al. 1997; Alessandri et al. 2013) (Fig. 2b). Because large spheroids (≳ 500 µm) exhibit a strong oxygen gradient with a hypoxic core, it was unclear if solid stresses alone or the combination of solid stress and hypoxia inhibited growth (Däster et al. 2017; Rodrigues et al. 2021). To avoid hypoxic effects, spheroids were confined within small alginate microcapsules, ensuring optimal penetration of oxygen throughout the whole aggregate (Alessandri et al. 2013; Le Maout et al. 2020). The effect of the capsule-imposed confinement depended on the confluency (i.e., cell density) of the aggregates, which increased concomitantly with aggregate growth. In a pre-confluent state where the aggregate volume was not yet limited by confinement, the aggregate growth regime resembled the exponential growth of freely growing aggregates (Alessandri et al. 2013; Le Maout et al. 2020). This correlated with minimal compressive stresses, derived from strain measurements, and resulted in uniform cell volume and cell proliferation along the spheroid radius (Montel et al. 2012; Dolega et al. 2017; Han et al. 2020). Conversely, in a post-confluent state, characterized by a notable increase in cell mass fraction and a compressive stress gradient, cell aggregates exhibited near growth arrest with minimal residual growth occurring at the periphery (Montel et al. 2012; Delarue et al. 2014; Dolega et al. 2017; Le Maout et al. 2020). Quantification of mechanical stresses up to 5 kPa in 3D aggregates confirmed the radial gradient of stress and its inverse correlation with cell proliferation (Montel et al. 2012; Dolega et al. 2017). In sum, the compressive stresses resulting from ECM confinement on expanding aggregates inhibited cell proliferation in tumor spheroids, independent of oxygen supply (Fig. 2b) (Dolega et al. 2017; Han et al. 2020; Mahajan et al. 2021).

The level of confinement and compressive stresses imposed on growing tumor spheroids can be partially released in viscoelastic or proteolytically degradable hydrogels (Wisdom et al. 2018; Taubenberger et al. 2019; Chaudhuri et al. 2020; Mahajan et al. 2021). In degradable matrices, spheroid growth was still limited in a matrix-stiffness-dependent manner, albeit to a lesser level than in non-degradable hydrogels (Taubenberger et al. 2019; Mahajan et al. 2021). Measuring compressive stresses acting on spheroids showed that the magnitude of stresses is more stiffness-dependent in degradable matrices (*σ* = 0.5 kPa in soft hydrogels compared with *σ* = 1–1.5 kPa in stiff hydrogels) (Mahajan et al. 2021). In contrast, in non-degradable matrices, the measured compressive stresses (*σ* ~ 2 kPa) did not notably change with substrate stiffness. Overall, the spheroid growth in these confinement conditions correlated with compressive stresses, modulated by both matrix stiffness and degradability.

The gradient of compressive stresses towards the core of growing tumor cell aggregates is thought to result in an opposite gradient in cell volume, which is typically smaller towards the core (Fig. 2b) (Han et al. 2020; Kang et al. 2021; Chang et al. 2024). To study the role of intratumoral stresses in controlling cell volume, confinement was released by enzymatic digestion of the scaffold. Six hours after release of confinement, cells in the aggregate core adapted and showed an increased volume. The initial cell volume gradient became much weaker, driven by swelling of cells in the core, and shrinking of cells at the periphery, suggesting supracellular fluid exchanges following stress release (Han et al. 2020). Such volume gradients in multicellular aggregates have been attributed to fluid flow through gap junctions, which are intracellular cell–cell connections (Wade et al. 1986; Teleki et al. 2014). Inhibition of gap junctions in tumor cell aggregates abolished the differences in nuclear and cellular volume across the growing aggregates, from the core to the periphery (Han et al. 2020; Chang et al. 2024), and additionally delayed the progression to an invasive phenotype (Han et al. 2020). Thus, confinement and compressive stresses not only impact single cell volume changes (through stretch-activated ion channels), but also collective regulation of cell and nuclear volume (through gap junctions). We conclude that activation of volume-regulating pathways is essential in controlling cell differentiation, proliferation, and tumor invasiveness.

## Adaptive mechanisms under high levels of confinement

### Adaptation to high confinement by nuclear sensing, mitochondrial repositioning, and amoeboid migration

We have until now considered limitations to cell volume, and stress-induced volume gradients in aggregates, but without considering more important cellular deformations. Under high levels of confinement, such as migration through constrictions, cells can be subjected to large deformations that indent or compress the cytoplasm and organelles, without necessarily changing cell or nuclear volume (Fig. 1). We here detail a collection of recent studies that investigated in detail how organelles, in particular the nucleus and the mitochondria, sense and respond to high levels of confinement in vitro, and in the case of tumor cells, how they adapt cell mechanics to facilitate cell migration and invasion into the stroma.

To understand how cells, and nuclei in particular, sense confinement, several groups have used planar confinement devices to limit cell and nuclear height (Le Berre et al. 2014; Liu et al. 2015; Lomakin et al. 2020; Venturini et al. 2020). Their work has unveiled an adhesion-independent pathway triggering increased actomyosin contractility under confinement. Large deformations of the nucleus led to unfolding of the inner nuclear membrane, triggering calcium release from intracellular calcium stores into the cytoplasm (Lomakin et al. 2020; Venturini et al. 2020). Calcium release allowed for the Ca^2+^ cytosolic phospholipase A2 (cPLA_2_) to be recruited to the nuclear envelope, where it catalyzed the formation of arachidonic acid (AA). AA and increased cytosolic calcium both increased cell contractility by promoting myosin activity through the RhoA pathway (Katayama et al. 2010; Brown et al. 2014; Lomakin et al. 2020; Venturini et al. 2020). This increase in cell contractility triggered a switch to fast amoeboid migration (Brown et al. 2014), which could be a mechanism in vivo to enable migration in confined spaces and escape from confined areas (Fig. 2c). For a more extensive review on cell migration in confined 3D environments, we direct the reader to other recent reviews on cell migration and cancer cell metastasis (McGregor et al. 2016; Paul et al. 2017; Yamada and Sixt 2019; Cambria et al. 2024; Lee and Holle 2024).

Cells under high levels of confinement show signs of adapting to it. Acute confinement of HeLa cells to 3–7 µm height led to an accumulation and immobilization of mitochondria and the endoplasmic reticulum within nuclear indentations (Ghose et al. 2023; Liu et al. 2024b). The functional consequences were a surge in nuclear ATP production, which in turn led to an increase in chromatin accessibility and faster repair of DNA damage (Ghose et al. 2023). Interestingly, mitochondrial relocalization under mechanical confinement had also been observed in cancer cell invasion and migration (Desai et al. 2013; A. Mosier et al. 2024; Liu et al. 2024b). The mitochondrial positioning appears to depend on the level of confinement, the engagement of adhesions, and the migration mode. In adhesion-based confined migration (7–15 µm), anterior positioning of mitochondria correlated with increased cell velocity (Desai et al. 2013; A. Mosier et al. 2024). High confinement combined with low adhesion triggered a mesenchymal-to-amoeboid transition (Liu et al. 2015). In amoeboid migration under 3 µm confinement, the mitochondria were found predominantly in the posterior part of the cell, resulting in a localized ATP production in the energy-intensive regions of stable bleb formation (Liu et al. 2024b) (Fig. 2c). Cells therefore have mechanisms to respond and adapt to confinement-induced stress in an energy-efficient manner by relocating ATP production (Fig. 2c).

### Confinement influences individual and collective cancer cell migration

Cell migration can be a strategy for cells to escape confinement and, in the case of tumors, also drives tumor invasion. Tumor cell invasion into the surrounding matrix depends on cell mechanical properties, the properties of the surrounding matrix, and their interaction (Lautscham et al. 2015; Ilina et al. 2020; Kang et al. 2021). However, it is not obvious how confinement and the associated mechanical stresses influence tumor invasion. To elucidate the effect of compressive stresses on cell mechanics, atomic force microscopy (AFM) measurements were performed on cancer cells migrating in 2D from an unconfined environment to a confined space. With increasing confinement cancer cells became softer and altered their shape (Rianna et al. 2020; Roberts et al. 2021). These changes were directly correlated with increased actin density at the lateral cell wall and with cytoplasmic YAP localization (Elosegui-Artola et al. 2017; Rianna et al. 2020). These findings highlight that cell softening and YAP activity inherently depend on confinement, and provide cells with a mechanoadaptive mechanism that facilitated migration through narrow spaces.

The behavior of 3D cell aggregates was slightly different from single cells. AFM measurements of whole tumor spheroids and the individual cells immediately after isolating them from the surrounding ECM revealed that both aggregate and cell stiffness (at least for those cells on the aggregate periphery) increased with the stiffness of the confining ECM (Taubenberger et al. 2019). A similar positive correlation between tumor spheroid stiffness and ECM stiffness was found in non-invading aggregates, measured by Brillouin microscopy, and optical tweezers (Taubenberger et al. 2019; Han et al. 2020; Mahajan et al. 2021). In aggregates with an invasive phenotype, the cells at the periphery were softer than the core but became stiffer with higher compressive stresses (Han et al. 2020; Mahajan et al. 2021). The difference between cell response to 2D and 3D confinement could be due to the associated high compressive stresses and to the corresponding volume gradient observed in 3D confinement.

When the cell volume of tumor cell aggregates was manipulated by osmotic pressure, only the peripheral cells showed changes in volume and cell stiffness, without any observed impact on tumor spheroid growth (Han et al. 2020). Compression by osmotic pressure led to decreased cell volume, increased stiffness, and lower invasiveness (Han et al. 2020), while conversely, cell swelling showed opposite trends. Overall, cell invasiveness consistently correlated with cell volume and inversely correlated with cell stiffness. In sum, 3D confinement of cell aggregates tends to decrease invasiveness due to the compressive stresses and associated effects on cell volume, at least in non-malignant cells.

Cancer cell migration is also often studied as collective behavior (Ilina et al. 2020; Kang et al. 2021). In tumor spheroids, high compressive stresses tend to maintain cells in a solid-like or *jammed* state, which is with no or little cell movement and no changes in cell neighbors. At the spheroid periphery, local variations in cell–cell interactions can trigger an *unjamming* transition to a more fluid-like state where there is lower coordination between cell velocities and cells can change neighbors (Ilina et al. 2020; Kang et al. 2021). Both jammed and unjammed protrusions from tumor spheroids have been observed and thus can lead to different modes of tumor invasion (Ilina et al. 2020; Kang et al. 2021). Notably, the transition between the jammed and unjammed states is highly dependent on the density of the surrounding matrix and therefore on the confinement conditions. High matrix density will promote a jamming transition, while low matrix density will promote an unjamming to a liquid-like or even more sparse gas-like state in highly invasive spheroids (Kang et al. 2021).

These observations on tumor cell softening and tumor invasion mode are dependent on cell type (cancer cell malignancy) and matrix density, typically given by the concentration of collagen used in many cancer cell migration studies. The question that remains open, however, is how firmly the observations depend on matrix stiffness or on pore size. In collagen matrices, it is difficult to decouple stiffness from pore size. Nonetheless, there are other hydrogel matrices, such as alginate, which would enable the investigation of the effect of stiffness in two parameters independent of each other. Investigating tumor invasion in a variety of engineered matrices will help understand how different tissue and confinement contexts influence the risk of invasion.

## Confinement restricts macromolecular transport in the cellular microenvironment

In the first three sections, we focused on intracellular effects of cell confinement. We now turn our attention to the immediate cellular microenvironment, which is also impacted by confinement. Even low levels of confinement can modify tissue homeostasis by creating a physical barrier to the diffusion of macromolecules and vesicles, on top of restricting the movement of cells. It is indeed a recognized function of the ECM to serve as a physical barrier and anchorage point for tissues (Humphrey et al. 2014). However, this aspect of the ECM is often ignored in in vitro tissue models.

### ECM deposition is enhanced under macromolecular crowding conditions

**Fig. 3:**
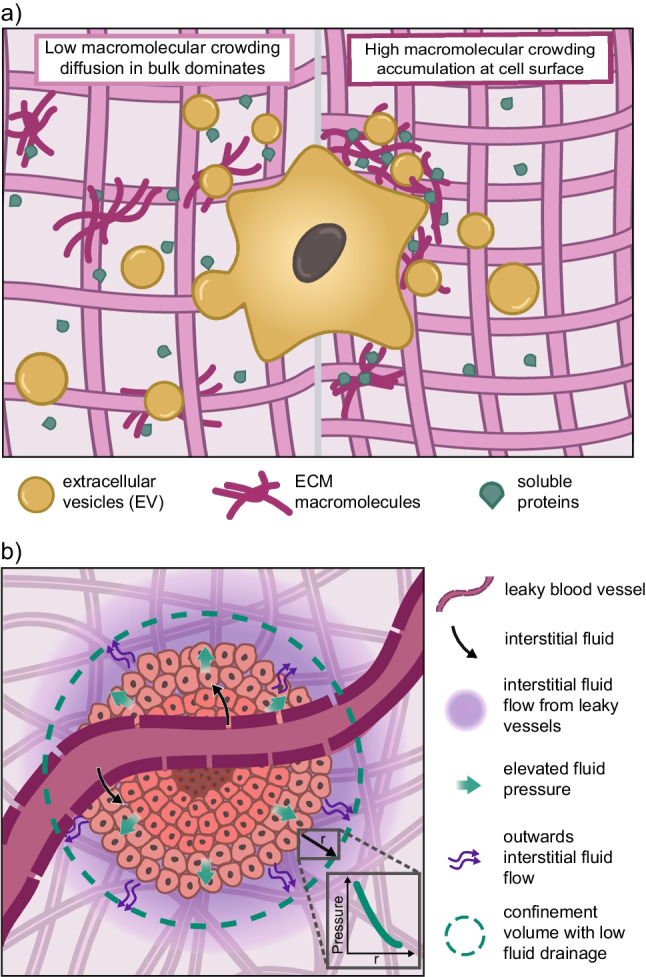
Limitations to transport under ECM-induced confinement. **a** Polymer density in the ECM is important in determining the range of transport of EVs and the diffusion of macromolecules. Under high polymer density/macromolecular crowding (right), large EVs are trapped, deposited ECM molecules remain at the cell surface facilitating assembly, and soluble proteins diffuse at the cell surface rather than in the bulk. **b** In tumor spheroids, ECM density creates a boundary layer for the tumor. The fluid coming from leaky blood vessels leads to a build-up of fluid pressure, but the confinement volume prevents the escape and drainage of the fluid. This leads to an interstitial fluid pressure gradient (green, solid line) in the immediate tumor surrounding. The pressure gradient drives fluid flow away from the tumor center, reducing accessibility to the tumor for soluble drugs

There is strong evidence that the barrier formed by the ECM in vivo, or by ECM mimics in vitro, is an essential regulator of cellular communication and indirectly of cell and tissue function (Nelson and Bissell 2006; Bissell and Hines 2011). A dense ECM matrix can act as a barrier in two directions (Fig. 3a). First, it limits the molecular diffusion from the extracellular space to the pericellular space. This is seen in the limited drug accessibility to tissues and tumors that have a thick ECM layer (or capsule) around them (Li and Kumacheva 2018; Souri et al. 2022; Narciso et al. 2023). Second, it also prevents the diffusion into the extracellular space of cell-secreted compounds, retaining them in the pericellular space. The net effect of this restricted diffusion is to accumulate macromolecules and enzymes at the cell surface (Chapanian et al. 2014; Li and Mooney 2016; Axpe et al. 2019; Raghunath and Zeugolis 2021). This local accumulation of macromolecules constitutes another type of confinement, at the molecular level, which is similar to macromolecular crowding.

Macromolecular crowding is a well-described physical phenomenon that originates in the simple fact that two molecules cannot occupy the same physical space (excluded volume effect) (Zhou et al. 2008). In tissues, the macromolecule concentration is typically > 80 mg/ml (in blood) and ≥ 400 mg/ml in the cytoplasm. Conversely, typical media used for in vitro cell culture has a protein concentration that can vary between 5 and 50 mg/ml (based on the amount of serum used). To mimic the typical crowding of tissues in vivo, a high concentration of polymers in solution can be used as crowding agents. Crowding agents have been shown to increase ECM deposition and assembly at the cell surface in vitro (Lareu et al. 2007; Chen et al. 2011; Benny et al. 2015; Raghunath and Zeugolis 2021; Liu et al. 2024a). Increased ECM deposition is thought to result not from higher synthesis, but instead from volume exclusion, whereby macromolecular crowders restrict the diffusion of matrix proteins and of matrix remodeling enzymes into the bulk media volume (Fig. 3a). For example, restricted diffusion of pro-collagen fibers and of pro-collagen proteinases (that cleave the N- and C-terminal ends of the pro-collagen fibrils) enhanced the conversion of pro-collagen to collagen and thereby accelerated collagen fibril assembly (Chen et al. 2011). Deposition of other ECM proteins, such as fibronectin and various types of collagens, was similarly increased under macromolecular crowding conditions (Benny et al. 2015; Liu et al. 2024a). Such macromolecular crowding strategies have been abundantly used in tissue engineering approaches to accelerate ECM deposition and assembly (Chen et al. 2011).

Given how polymers in solution act by limiting diffusion of ECM molecules and processing enzymes, it is reasonable to expect a physical, cross-linked polymer-based matrix or matrix-mimic to have a similar effect. ECM protein deposition and assembly has indeed been observed around 3D encapsulated cells across a variety of hydrogel types (Loebel et al. 2019; Krattiger et al. 2024) and cell types (Loebel et al. 2019; Jain et al. 2023; Eiken et al. 2024). For organoids, the local matrix deposition enabled by the presence of a diffusion barrier had important functional consequences: if basement membrane proteins are deposited, these serve as a basal polarity signal to cells, a prerequisite for epithelial sheet polarization and lumenization, as is typically observed in Matrigel organoid cultures (Jain et al. 2023; Chang et al. 2024).

The physical properties of the engineered matrices are likely to be important for macromolecular crowding effects and nascent ECM protein deposition, although further research is needed on this matter. It was observed that increasing the stiffness of the hydrogel decreased the thickness of the deposited protein layer, suggesting that the density of the surrounding hydrogel does further impact ECM deposition (Loebel et al. 2019). There is likely a trade-off between increasing ECM accumulation and limiting cell function, whereby low levels of confinement lead to an increase in ECM accumulation immediately around cells, whereas a higher level of confinement would lower cell secretion and ECM deposition, through other yet unknown mechanisms. We can speculate that the ability for cells to dynamically alter their shape and membrane tension could impact the rate of exocytosis and ECM secretion. Furthermore, high levels of confinement could ultimately prevent assembly of ECM macromolecules. The density of the local matrix and its stress-relaxing properties (discussed above) could therefore have a strong influence on matrix deposition and assembly. We also note that in conditions favorable to high ECM deposition, higher deposition could locally increase the level of confinement, leading to indirect effects on ECM deposition and macromolecule diffusion within the extracellular space.

### Vesicle transport is partially dependent on ECM density and mechanics

Emerging evidence suggests that confinement can also impact long-range cellular communication by regulating the diffusion of large proteins and of extracellular vesicles (EVs) carrying cell-secreted macromolecules. The limitation on macromolecule and EV diffusion will depend on one hand on their own size and on the other hand on pore size (and therefore on matrix or polymer density). Small molecules whose hydrodynamic radius is much smaller than pore size will diffuse unhindered (Fig. 3a). Proteins and vesicles whose size is on the order of pore size will exhibit anomalous diffusion, while larger ones will be significantly slowed down, or even immobilized (Lustig and Peppas 1988; Masuda et al. 2005; Banks and Fradin 2005; Kihara et al. 2013; Li and Mooney 2016; Axpe et al. 2019). EVs, typically 50–500 nm, can be smaller or larger than pore size, depending on tissues or on the porosity of 3D engineered matrices. Surprisingly, even larger EVs are still able to be transported through ECMs, including passing basement membranes, although the mechanisms are not yet fully understood (Lenzini et al. 2020). Passive EV transport in confined conditions was found to be facilitated in stiff, stress-relaxing matrices, and reliant on adjustable water content in the EVs through aquaporins (Lenzini et al. 2020). In addition to these mechanisms, EVs can contain integrins, matrix metallo-proteinases, and ECM cross-linking enzymes that enable them to interact with ECM, facilitating either their local concentration or transport and passage of barriers such as the basement membrane (van Niel et al. 2022; Sariano et al. 2023). Due to these numerous EV–ECM interactions, it is difficult to decipher exactly how much the ECM properties and the confinement conditions limit EV transport and dissemination in tissues. Given the recently discovered importance of EVs in proximal and long range cellular communication, including in cancer, it will be important to design new assays to understand the role of confinement on EV-mediated communication (van Niel et al. 2022; Nishida-Aoki and Ochiya 2024).

### Confinement-induced fluid pressure gradient restricts fluid transport to the tumor

Interstitial fluid pressure (IFP) has been identified early on as a biochemical abnormality in tumors (Young et al. 1950; Boucher et al. 1990). IFP is the isotropic stress exerted by the fluid within the interstitial space, exerted equally in all directions (Stylianopoulos et al. 2018). In normal tissue, fluid homeostasis is maintained with near-zero IFP, but in tumors, this balance can be disrupted, leading to elevated IFP levels ranging from 1 to 5 kPa. The accumulation of interstitial fluid in the tumor interstitium is a result on one hand of leaky tumor blood vessels and on the other hand of a dense ECM and deficient lymphatic drainage (Stylianopoulos et al. 2013, 2018; Salavati et al. 2022). A steep IFP gradient at the tumor boundary drives interstitial fluid flow from the tumor outward towards the surrounding host tissue (Fig. 3b). While the literature is clear about the steep gradient at the tumor periphery, the exact distribution of IFP within tumors, whether uniform or gradient, remains debated (Baxter and Jain 1989; Stylianopoulos et al. 2018; Hansem et al. 2019; Follain et al. 2020; Nia et al. 2020; Waldeland et al. 2021; Salavati et al. 2022). Although not all contributing factors to IFP are known, IFP is closely correlated with matrix properties, such as stiffness and density, and thus with the level of confinement of tumors (Fovargue et al. 2020; Salavati et al. 2022).

IFP is used as a biomarker for identifying tumor malignancy and assessing treatment response (Islam et al. 2021; Salavati et al. 2022; Kim et al. 2023; He et al. 2024). One reason is that IFP has been identified as a major obstacle to the effective delivery of therapeutic agents to target sites, both systemically and locally (Heldin et al. 2004; Steuperaert et al. 2019). Firstly, because an elevated IFP impedes convection-driven drug transport from the blood to the interstitium (Jain et al. 2014). This limits the primary transport mechanism to diffusion, which is slow. Secondly, the steep pressure gradient at the tumor edge will further limit drug access to the tumor site. Confinement imposed by a dense ECM further exacerbates this effect, hindering the diffusion of large macromolecules and nanoparticles (Baish et al. 2011; Miao et al. 2015; Dewhirst and Secomb 2017). Simulations indicate that compound delivery is generally limited to a zone of around 100 μm surrounding tumor blood vessels (Hoffmann et al. 2020).These findings underscore the crucial role of IFP, in association with confinement, in tumor treatment due to limitations to macromolecular transport. IFP can also have a direct impact on tumor progression and metastasis, and this is discussed elsewhere (Lunt et al. 2008; Stylianopoulos et al. 2013, 2018). Overall, these findings position IFP as a key focus for future research and therapeutic interventions, as modification and monitoring can be an indicator of enhanced tumor tissue penetration and overall drug efficacy (Mohammadabadi et al. 2020; Wu et al. 2021; Yu et al. 2021)].

## Conclusion and outlook

Whether in tissues or in engineered 3D tissue models, cells are subjected to various levels of confinement. We have divided these confinement levels into *low level* (limitation to cell volume and cell volume changes), *intermediate level* (combination of high cell and ECM density), and *high level* (deformation of cell and nucleus). We saw throughout this review that each of these levels of confinement has different implications for cell function and activation of mechanosensitive pathways. At low levels of confinement, the confinement volume itself is not the critical factor for maintaining cell viability, rather the degree of mechanoactivation is. Mechanoactivation itself will depend on the properties of the engineered niche, such as its stiffness and stress relaxation properties. Further dependencies on cell type will have to be individually tested, as for some cell types, confinement led to a clear impact in cell function, such as the mitigated inflammatory response in macrophages. Many studies report that activation of the cytoskeleton (actin protrusions and contractility) and engagement of focal adhesions are essential for cell viability, as is YAP/TAZ activation, and these partly depend on scaffold stiffness. We also found that in confining elastic matrices, the level of YAP may not be sufficient to drive stem cell differentiation, which would require relieving the confinement. Viscoelastic matrices can provide this confinement relief, as fast-relaxing hydrogels allowed for greater cell volume expansion and cell shape changes through activation of stretch-activated channels. Other matrices that undergo reorganization at the molecular scale also allowed for confinement relief, and the subsequent fast (seconds) cellular deformations showed similar benefits in terms of enhancing hMSC differentiation. Why short-term stress relaxation and cell movements have such an impact on long-term differentiation of hMSCs and organoids requires further investigation. One potential pathway could be activation of the cytosolic phospholipase cPLA2/AA pathway. This pathway is activated by stretching or unfolding of the nuclear membrane, as observed under high confinement levels, but may be already activated by lower levels of confinement and smaller cell deformations. Further studies must determine if stress relaxation and cell volume expansion impact this cPLA2/AA pathway as well, possibly downstream of stretch-activated ion channels. Further effects of high levels of confinement included mitochondrial repositioning as well as localized ATP production to power DNA repair and amoeboid migration. These indicate that cells have multiple strategies to adapt to confinement conditions and that mitochondria may be particularly susceptible to confinement. Given the known crosstalk between mechanotransduction and metabolism (Romani et al. 2021), it would be interesting to investigate if confinement-induced mechanotransduction lead to further changes in metabolic functions or mitochondrial dynamics.

We focused on tumor spheroids, because 3D in vitro tumor models are increasingly used to understand cancer biology and for drug testing. The level of confinement in tumor spheroids depends not only on ECM density but also on cell density and therefore on tumor growth. Under intermediate confinement, the emergence of a gradient of compressive stresses exerted strong constraints on cell volume, limiting cell proliferation. In 3D tumor spheroids, these compressive stresses also limited cell softening and invasiveness. However, because matrix density and confinement can also impact the mode of invasion through a *jamming* or *unjamming* transition, the overall effect of confinement on tumor progression and invasion remains unclear. Further investigations will have to determine how the pore size and stiffness of the surrounding matrix impact tumor invasion and if the epithelial-to-amoeboid transition observed in single cells also occurs in cell aggregates.

We lastly covered how confinement leads to restrictions to transport in the cellular microenvironment. The size-selective barrier created by a dense ECM has implications for molecular and vesicular transport and for cellular paracrine communication. Here, the difficulty to differentiate between active transport (involving binding to the ECM of macromolecules and vesicles) and passive transport driven by diffusion or convection makes it difficult to draw a strong conclusion. Further studies, for example, using inert polymers as a cell scaffold may help to tease these apart. Beyond the simple limitation to diffusion, a dense extracellular space could have further *crowding* effects. We briefly mentioned how polymers in solution (“crowding agents”) lead to increased ECM deposition, but also increased ligand binding at the cell surface (Chapanian et al. 2014). Intracellular macromolecular crowding in the cytoplasm and nucleus is known to change binding affinities by several orders of magnitude (Zhou et al. 2008; Hancock, Ronald and Jeon, Kwang W. 2014; Huet et al. 2014; Weiss 2014; Tsiapalis and Zeugolis 2021). There is some evidence that crowding at the cell surface induced by high concentration of soluble ligands, by long, membrane-tethered proteoglycan chains (*the glycocalyx*) can impact oligomerization of receptors (Needham et al. 2016; Kuo et al. 2018). Whether the ECM itself could lead to such excluded volumes effects in the extracellular space, and impact not only cell surface binding events, but also the activation of mechanosensitive pathways remains to be determined. Note that the level of macromolecular crowding (concentration of macromolecules) is certainly lower in the extracellular space compared with the intracellular space, but the ECM and glycocalyx together are composed of long polymer chains that could occupy much larger volumes than intracellular molecules.

In each of the topics discussed, from the activation of mechanosensitive pathways to driving cell differentiation, nascent ECM deposition or vesicular transport, we indicated when relevant the matrix properties that have an influence. As new strategies to engineer matrices with macroporosities emerge, it will become possible to further modulate the level of confinement. It will be interesting to investigate the relative influence of pore size compared with other properties, such as stress relaxation. It is also obvious that 2D and 3D mechanobiology diverge in the effects of stresses on cells. The combination of confinement with other active mechanical stressors (e.g., stretch) could also lead to different responses in 3D confined environments compared with 2D environments. We hope that these questions of how confinement modulates other mechanobiology pathways in healthy and diseased states will inspire future research directions.

## Data Availability

No datasets were generated or analysed during the current study.
